# Glimepiride Reduces the Expression of PrP^C^, Prevents PrP^Sc^ Formation and Protects against Prion Mediated Neurotoxicity

**DOI:** 10.1371/journal.pone.0008221

**Published:** 2009-12-09

**Authors:** Clive Bate, Mourad Tayebi, Luisa Diomede, Mario Salmona, Alun Williams

**Affiliations:** 1 Department of Pathology and Infectious Diseases, Royal Veterinary College, North Mymms, United Kingdom; 2 Department of Molecular Biochemistry and Pharmacology, Istituto di Ricerche Farmacologiche “Mario Negri,” Milan, Italy; University of North Dakota, United States of America

## Abstract

**Background:**

A hallmark of the prion diseases is the conversion of the host-encoded cellular prion protein (PrP^C^) into a disease related, alternatively folded isoform (PrP^Sc^). The accumulation of PrP^Sc^ within the brain is associated with synapse loss and ultimately neuronal death. Novel therapeutics are desperately required to treat neurodegenerative diseases including the prion diseases.

**Principal Findings:**

Treatment with glimepiride, a sulphonylurea approved for the treatment of diabetes mellitus, induced the release of PrP^C^ from the surface of prion-infected neuronal cells. The cell surface is a site where PrP^C^ molecules may be converted to PrP^Sc^ and glimepiride treatment reduced PrP^Sc^ formation in three prion infected neuronal cell lines (ScN2a, SMB and ScGT1 cells). Glimepiride also protected cortical and hippocampal neurones against the toxic effects of the prion-derived peptide PrP82–146. Glimepiride treatment significantly reduce both the amount of PrP82–146 that bound to neurones and PrP82–146 induced activation of cytoplasmic phospholipase A_2_ (cPLA_2_) and the production of prostaglandin E_2_ that is associated with neuronal injury in prion diseases. Our results are consistent with reports that glimepiride activates an endogenous glycosylphosphatidylinositol (GPI)-phospholipase C which reduced PrP^C^ expression at the surface of neuronal cells. The effects of glimepiride were reproduced by treatment of cells with phosphatidylinositol-phospholipase C (PI-PLC) and were reversed by co-incubation with p-chloromercuriphenylsulphonate, an inhibitor of endogenous GPI-PLC.

**Conclusions:**

Collectively, these results indicate that glimepiride may be a novel treatment to reduce PrP^Sc^ formation and neuronal damage in prion diseases.

## Introduction

The transmissible spongiform encephalopathies, otherwise known as prion diseases include Creutzfeldt-Jakob disease and kuru in humans, as well as important livestock diseases such as scrapie in sheep and goats and bovine spongiform encephalopathy in cattle. The central event in these diseases is the conversion of a host encoded cellular prion protein (PrP^C^) into abnormally folded, disease-associated isoforms (PrP^Sc^) in the brains of infected animals [1]. Although the primary amino acid sequence remains the same, during the conversion process a portion of the α-helix and random coil structure in PrP^C^ is refolded into a β-pleated sheet in PrP^Sc^ [2]. This change in secondary structure is accompanied by changes in the biological and biochemical properties of the PrP^Sc^ protein, including reduced solubility and an increased resistance to proteases [3]. Consequently, aggregates of PrP^Sc^ accumulate in association with neurones in affected brain areas [4], a process which is thought to lead to synapse degeneration and ultimately neuronal death. PrP^Sc^ is believed to constitute the major and perhaps only component of the infectious particle [5]. While the correlation between PrP^Sc^ and infectivity is not completely clear [6], cell based studies routinely measure the amount of PrP^Sc^ as an indicator of infectivity.

The production of PrP^Sc^ and the progression of prion diseases are dependent upon the presence of PrP^C^ [7], [8], [9]. PrP^C^ is linked to the membrane by a glycosylphosphatidylinositol (GPI) anchor [10] and can be released from the surface of cells by treatment with phosphatidylinositol-phospholipase C (PI-PLC) [11]. Treatment of prion-infected neuronal cells with PI-PLC reduced PrP^Sc^ formation [12], [13] indicating that PrP^Sc^ is formed from PrP^C^ expressed at the cell surface, or from PrP^C^ that had been expressed at the cell surface. This conclusion is supported by observations that PrP^C^ reactive antibodies reduced PrP^Sc^ formation in prion-infected neuronal cells [14], [15]. Thus, any treatment that affected the amount of PrP^C^ expressed at the cell surface may also be expected to affect PrP^Sc^ formation.

Glimepiride is a sulphonylurea used to treat non insulin-dependent diabetes mellitus. It some cells it activates an endogenous GPI-PLC [16], [17]. Thus, in adipocytes glimepiride treatment released some GPI-anchored proteins from surface membranes [18], [19] and caused the redistribution of other GPI-anchored proteins [20]. We therefore investigated the effect of glimepiride treatment on the amount of PrP^C^ at the surface of primary cortical neurones and prion-infected neuronal cell lines. Glimepiride treatment reduced the amount of PrP^C^ at the surface of neuronal cell lines and primary cortical neurones. The effects of glimepiride were similar to the effects of PI-PLC; both caused the release of a soluble, deacylated PrP^C^. Treatment with glimepiride also reduced PrP^Sc^ formation in 3 prion-infected neuronal cell lines (ScGT1, SMB and ScN2a cells). In addition, the effects of glimepiride treatment on prion neurotoxicity were examined. PrP^C^ is required for the neurotoxicity of PrP^Sc^ [21] and the process of prion-induced neurodegeneration is commonly examined by incubating neurones with either recombinant PrP or specific PrP-derived peptides. A synthetic peptide containing amino acids 82 to 146 of the human PrP protein (PrP82–146) corresponding to a major PrP fragment isolated from the brains of patients with Gerstmann-Sträussler-Scheinker disease (GSS) [22], was toxic to cultured cortical neurones [23], [24]. Here we report the effects of glimepiride on PrP82–146 induced activation of cytoplasmic phospholipase A_2_ (cPLA_2_) and neuronal survival.

## Methods

### Cell lines

Prion-infected neuronal cell lines (ScGT1, ScN2a and SMB cells) [25], [26], [27] were grown in Ham's F12 medium supplemented with 2 mM glutamine, 2% foetal calf serum (FCS) and standard antibiotics (100 U/ml penicillin and 100 µg/ml streptomycin). Cells were plated in 6 well plates (10^5^ cells/well) and allowed to adhere overnight before the addition of test compounds. The medium was changed twice daily and the amount of cell-associated PrP^Sc^ measured after 7 days. Cells were washed twice in phosphate buffered saline (PBS) and homogenised at 10^6^ cells/ml in an extraction buffer containing 10 mM Tris-HCl, 100 mM NaCl, 10 mM EDTA, 0.5% Nonidet P-40 and 0.5% sodium deoxycholate. Nuclei and large fragments were removed by centrifugation (300×*g* for 5 minutes) and the supernatant digested with 5 µg/ml proteinase K for 1 hour at 37°C, digestion was stopped using mixed protease inhibitors (AEBSF, Aprotinin, Leupeptin, Bestain, Pepstatin A and E-46) (Sigma, Poole, UK). Culture supernatants were also collected to see if PrP^Sc^ was released from cells. They were digested with 5 µg/ml proteinase K for 1 hour at 37°C and stopped with mixed protease inhibitors (as above). In some studies cell extracts/supernatants were digested with 50 µg/ml thermolysin, which is reported to digest PrP^C^ without affecting protease sensitive PrP^Sc^ [28], [29]. The digested supernatant was concentrated by centrifugation with a 10 kDa filter (Sartorius vivaspin) and adjusted to an equivalent of 10^6^ cells/ml. Samples were heated to 95°C for 5 minutes and tested in a PrP specific enzyme-linked immunosorbent assay (ELISA). Uninfected N2a, GT1 or SMB-PS cells were used as controls.

### Primary neuronal cultures

Primary cortical neurones were prepared from the brains of mouse embryos (day 15.5) after mechanical dissociation [23]. Neuronal precursors were plated (2×10^5^ cells/well in 48 well plates pre-coated with 5 µg/ml poly-L-lysine) in Ham's F12 medium containing 5% FCS for 2 hours. Cultures were shaken (600 r.p.m for 5 minutes) and non-adherent cells removed by 2 washes in PBS. Neurones were subsequently grown in neurobasal medium (NBM) containing B27 components (Invitrogen, Paisley, UK) for 7 days. Immunolabelling studies showed that after 7 days cultures contained less than 5% glial cells (∼3% GFAP positive and less than 1% MAC-1 positive cells). Hippocampal neurones were prepared from the brains of adult mice as described [30]. Briefly, hippocampi were dissected from the adult brain tissue and triturated in Ham's F12 containing 5% FCS, 0.35% glucose, 0.025% trypsin, and 0.1% type IV collagenase (Invitrogen). After 30 minutes at 37°C, the cells were triturated with a 1 ml pipette and passed through a 100 µM cell strainer. Cells were washed twice in Ham's F12 medium containing 5% FCS and plated in 48 well plates pre-coated with 5 µg/ml poly-L-lysine (2×10^5^ cells/well) for 24 hours. Cultures were shaken (600 r.p.m for 5 minutes) to remove non-adherent cells, washed twice with PBS and cultured in NBM containing B27 components and 10 ng/ml glial-derived neurotrophic factor (Sigma) for 7 days. Neurones were subsequently pre-treated with test compounds (glimepiride, glipizide, glibenclamide, p-chloromercuriphenylsulphonate (p-CMPS) or PI-PLC derived from *Bacillus cereus*, obtained from Sigma) and washed before the addition of PrP peptides or further analysis. Stock solutions of drugs were prepared in di-methyl sulphoxide (DMSO) and diluted on the day of use, vehicle controls were equivalent dilutions of DMSO. The survival of neurones was determined 5 days later using 25 µM thiazlyl blue tetrazolium (MTT); neuronal survival was reported as a percentage of control, vehicle treated neurones.

### Cell extracts

After treatment, cells were washed twice in PBS and homogenised in an extraction buffer containing 10 mM Tris-HCl, 100 mM NaCl, 10 mM EDTA, 0.5% Nonidet P-40, 0.5% sodium deoxycholate and 0.2% sodium dodecyl sulphate (SDS) at 10^6^ cells/ml and nuclei and large fragments were removed by centrifugation (300×*g* for 5 minutes). Mixed protease inhibitors were added to cell extracts where appropriate.

### Measurement of cell surface PrP^C^

The amount of PrP^C^ expressed at the cell surface was determined by two methods. In the first, 10^6^ treated neurones were subsequently pulsed with PBS containing 50 µg/ml of membrane-impermeable sulfo-biotin-X-NHS (Pierce, Cramlington, UK) for 10 minutes. Cells were then washed 4 times with ice cold PBS containing 10% FCS to remove unbound biotin and the amount of biotinylated PrP^C^ measured in a modified ELISA. Maxisorb Immunoplates (Nunc, Roskilde, Denmark) were pre-coated with 10 µg/ml streptavidin (Sigma) and blocked with 10% milk powder. Samples were added for 1 hour and the amount of bound biotinylated PrP^C^ was determined by incubation with the PrP-specific mAb ICSM18, anti-mouse IgG-alkaline phosphate and 1 mg/ml 4-nitrophenyl phosphate. Absorbance was measured at 450 nm and the amount of biotinylated PrP^C^ was calculated by reference to a standard curve of biotinylated recombinant PrP (Prionics, Zurich, Switzerland). The second method involved incubating treated cells with 0.2 units of PI-PLC/10^6^ cells for 1 hour at 37°C. PI-PLC acts on the GPI anchored proteins including PrP^C^ at the cell surface. The amount of PrP^C^ released into the supernatant following PI-PLC digestion was measured by PrP ELISA.

### Reverse phase chromatography of PrP^C^

Supernatants from glimepiride treated primary cortical neurones were applied to C18 columns (Waters, Elstree, UK). For comparison, PrP^C^ from cell extracts and PrP^C^ that had been digested with PI-PLC (0.2 units/ml for 1 hour at 37°C) were also added to C18 columns. Proteins were eluted by reverse phase chromatography under a gradient of acetonitrile in water and 0.1% trifluoroacetic acid (TFA). Fractions were collected, lyophilised, solubilised in extraction buffer and tested in a PrP ELISA.

### PrP ELISA

The amount of PrP in cell extracts/supernatants was measured by ELISA as described [31], [32]. Maxisorb Immunoplates were coated with a PrP-specific mAb (ICSM18 which recognises amino acids 146 to 159 of murine PrP). Samples were applied and detected with biotinylated mAb ICSM35 (which recognises a region between amino acids 91 and 110). This was detected using extravidin-alkaline phosphatase and 1 mg/ml 4-nitrophenyl phosphate. Absorbance at 450 nm was measured on a microplate reader and the amount of PrP calculated by reference to a standard curve of recombinant murine PrP (Prionics); its limit of detection was 0.05 ng/ml.

The amount of PrP82–146 in cell extracts was also determined by ELISA. Maxisorb Immunoplates were coated with 0.5 µg/ml of mouse mAb 3F4 (reactive with residues 109–112 of human PrP (Abcam, Chandler's Ford, UK), this mAb does not bind to murine PrP [33]. Samples were applied and detected with biotinylated ICSM35 (D-gen), followed by extravidin-alkaline phosphatase and 1 mg/ml 4-nitrophenyl phosphate. Absorbance was measured on a microplate reader at 450 nm and the amount of PrP82–146 was calculated by reference to a standard curve of PrP82–146.

### Activated cPLA_2_ ELISA

The activation of cPLA_2_ is accompanied by phosphorylation of the 505 serine residue, which can be measured by phospho-specific antibodies. The amount of activated cPLA_2_ in cell extracts was measured by ELISA as described [32]. Nunc Maxisorb Immunoplates were coated with 0.5 µg/ml of mouse mAb anti-cPLA_2_, clone CH-7 (Upstate, Milton Keynes, UK) for 1 hour and blocked with 10% FCS. Samples were incubated for 1 hour and the amount of activated cPLA_2_ was detected using a rabbit polyclonal anti-phospho-cPLA_2_ (Cell Signalling Technology). Bound antibodies were then detected using biotinylated anti-rabbit IgG (Dako), extravidin-alkaline phosphatase and 1 mg/ml 4-nitrophenyl phosphate. Absorbance was measured at 450 nm and the amount of activated cPLA_2_ present calculated by reference to a standard curve, using nonlinear regression. Samples were expressed as “units cPLA_2_”, 100 units being defined as the amount of cPLA_2_ in 10^6^ untreated cortical neurones. A standard curve ranging from 100 to 1.56 units/well was prepared from this sample using doubling dilutions.

### PGE_2_ assay

The amount of PGE_2_ produced by cells was determined by using an enzyme-immunoassay kit (Amersham Biotech, Amersham, UK). The detection limit of this assay is 20 pg/ml.

### Peptides

Peptides containing amino acids 82 to 146 of the human PrP protein (PrP82–146) corresponding to a PrP fragment found in certain prion-infected human brains [22], and a control peptide (PrP82–146scrambled) were synthesised by solid-phase chemistry and purified by reverse-phase HPLC. Stock solutions were thawed on the day of the experiment and sonicated for 10 minutes before addition to cells.

### Statistical Analysis

Comparison of treatment effects was carried out using one and two way analysis of variance techniques as appropriate.

## Results

### Glimepiride reduced PrP^C^ at the surface of neurones

**Figure 1:**
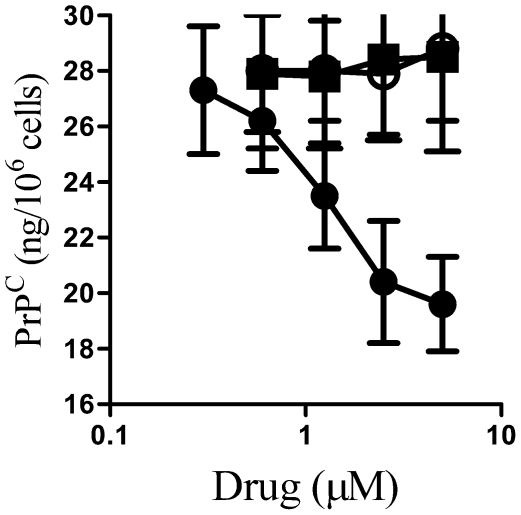
Glimepiride reduced neuronal PrP^C^ expression. The amount of PrP^C^ in whole cell extracts from primary cortical neurones treated for 1 hour with different concentrations of glimepiride (•), glibenclamide (○) or glipizide (▪) as shown. Values shown are the mean average amount of PrP^C^ in neuronal extracts (ng/10^6^ cells) ± SD, n = 12.

Since glimepiride has been reported to stimulate the release or redistribution of GPI-anchored proteins in adipocytes [17], [20], its effect on PrP^C^ in cortical neurones was examined. Treatment of neurones for 1 hour with glimepiride reduced the amount of PrP^C^ in cell extracts in a dose-dependent manner (Figure 1). This effect of glimepiride was not shared by glibenclamide or glipizide, two other sulphonylureas used to treat diabetes mellitus, which did not alter the PrP^C^ content of neurones. The reduction in the PrP^C^ content of neurones achieved following treatment with 5 µM glimepiride (19.6 ng/10^6^ cells±1.7 compared with 28.5±3.4, n = 12, P<0.01) was similar in magnitude to that observed in neurones treated with 0.2 units PI-PLC/ml (19.2 ng/10^6^ cells±2.8 compared with 28.5±3.4, n = 12, P<0.01), results consistent with the hypothesis that glimepiride activates an endogenous GPI-PLC. Higher concentrations of glimepiride or PI-PLC, or prolongation of treatment to 4 hours, did not cause any further reduction in the neuronal PrP^C^ content. Neither glimepiride nor PI-PLC affected the survival of neurones as measured by thiazyl blue tetrazolium.

**Figure 2:**
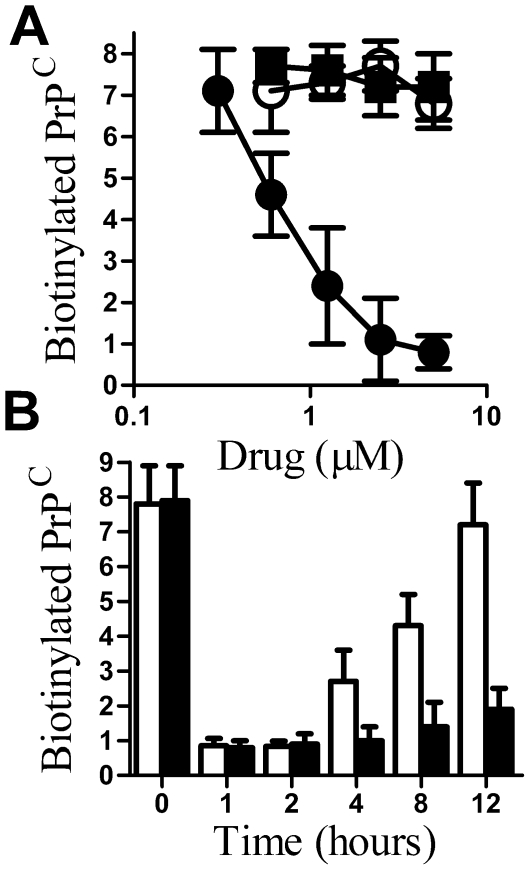
Glimepirde reduced the amount of PrP^C^ at the surface of neurones. (A) The amount of cell surface PrP^C^ on neurones treated for 1 hour with different concentrations of glimepiride (•), glibenclamide (○) or glipizide (▪) as shown. Values shown are the mean average amount of biotinylated PrP^C^ in cell extracts (ng/10^6^ cells) ± SD, n = 9. (B) The amount of cell surface PrP^C^ in cell extracts from cortical neurones taken at different time points after the addition of 5 µM glimepiride alone (*□*) or a mixture containing 5 µM glimepiride and 20 µg/ml cycloheximide (▪) as shown. Values shown are the mean average amount of biotinylated PrP^C^ in cell extracts (ng/10^6^ cells) ± SD, n = 9.

Cortical neurones treated with glimepiride for 1 hour were subsequently pulsed with a membrane-impermeable biotin ester to determine whether glimepiride affects the amount of PrP^C^ expressed at the cell surface. Treatment with glimepiride, but not glipizide or glibenclamide, reduced the amount of biotinylated PrP^C^ in cell membranes (Figure 2A). Treatment with 0.2 units PI-PLC/ml also reduced the amount of biotinylated PrP^C^ in cell membranes (0.6 ng/10^6^ cells±0.2 compared to 7.4±1.1, n = 9, P<0.01). Time course studies showed that the effect of glimepiride on the expression of PrP^C^ on the surface of cortical neurones was transient. Thus the amount of PrP^C^ labelled with cell-impermeable biotin in neurones pulsed with 5 µM glimepiride for 1 hour remained low for 2 hours after the cessation of glimepiride treatment and only returned to the levels seen in untreated cells after 12 hours (Figure 2B). When these experiments were conducted in the presence of 20 µg/ml cycloheximide, which inhibits the synthesis of proteins, the return of PrP^C^ to the cell surface was delayed, indicating that the PrP^C^ that appeared at the surface of glimepiride treated cells had been newly synthesised rather than rerouted from an existing intracellular pool.

### Glimepiride stimulated the release of PrP^C^ from neurones

**Figure 3:**
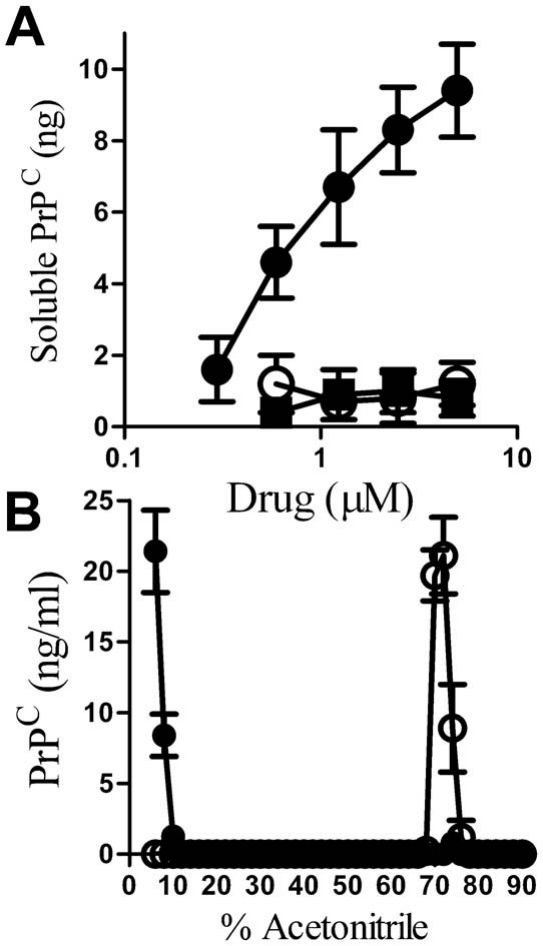
Glimepiride released PrP^C^ from cortical neurones. (A) The amount of PrP^C^ in the supernatant of cortical neurones treated for 1 hour with different concentrations of glimepiride (•), glibenclamide (○) or glipizide (▪). Values shown are the mean average amount of soluble PrP^C^ (ng/10^6^ cells) ± SD, n = 9. (B) The amount of PrP^C^ molecules eluted from C18 columns following elution with a gradient of acetonitrile in water containing 0.1% TFA. Fractions eluted from C18 columns loaded with whole cell extracts (○) or with supernatants from cortical neurones treated with 5 µM glimepiride (•). Values shown are the mean average amount of PrP^C^ (ng/ml) ± SD, n = 6.

Next we sought to determine if glimepiride stimulated the release of PrP^C^ from the cell by measuring the amount of PrP^C^ in the supernatants of cultured neurones. The addition of glimepiride for 1 hour, but not glibenclamide or glipizide, increased the amount of PrP^C^ released into cell supernatant (Figure 3A). Similarly, the addition of 0.2 units PI-PLC/ml for 1 hour increased the amount of PrP^C^ in the supernatants of neurones (9.9 ng/10^6^ cells±1.1 compared to 0.6±0.3 in untreated supernatants, n = 9, P<0.01). We noted that glimepiride treatment caused the release of specific GPI-anchored proteins. Thus, while digestion with 0.2 units PI-PLC/ml released both PrP^C^ and Thy-1 from neurones, treatment with 5 µM glimepiride released PrP^C^ but did not release Thy-1 (data not shown).

**Figure 4:**
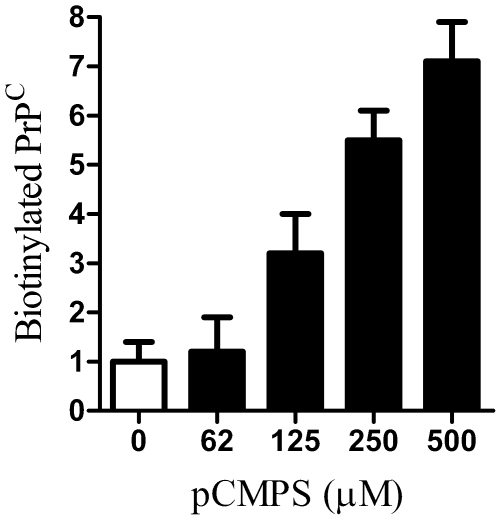
Glimepiride induced release of PrP^C^ is reversed by p-CMPS. The amount of PrP^C^ at the surface of cortical neurones treated for 1 hour with 5 µM glimepiride alone (□) or with combinations containing 5 µM glimepiride and different concentrations of p-CMPS as shown (▪). Values shown are the mean average amount of biotinylated-PrP^C^ (ng/10^6^ cells) ± SD, n = 8.

PrP^C^ can be released from cells by different mechanisms [11], [34], [35], [36], [37]. Therefore we sought evidence that the PrP^C^ in the supernatant of glimepiride treated cells was released following its digestion by a GPI-PLC. When cell-associated PrP^C^ containing an intact GPI anchor was bound to a C18 column and exposed to acetonitrile:water gradients, it was found in those fractions containing 70–76% acetonitrile. In contrast, cell-associated PrP^C^ molecules that had been digested by 0.2 units PI-PLC/ml did not bind to C18 columns. The PrP^C^ in supernatants from glimepiride treated neurones did not bind to C18 columns indicating that these PrP^C^ molecules had lost their hydrophobic acyl chains, consistent with the view that they had been digested by GPI-PLC (Figure 3B). Next we sought to determine if the effect of glimepiride could be reversed by the addition of p-CMPS, which inhibited GPI-PLC [38]. Whereas treatment with as much as 500 µM p-CMPS alone had no detectable effect on cell surface PrP^C^ (not shown), addition of p-CMPS to cortical neurones treated with 5 µM glimepiride increased the amount of PrP^C^ at their surface to control levels (Figure 4), confirming that glimepiride activates an endogenous GPI-PLC.

### Glimepiride reduced the PrP^Sc^ content of prion infected neuronal cells

**Figure 5:**
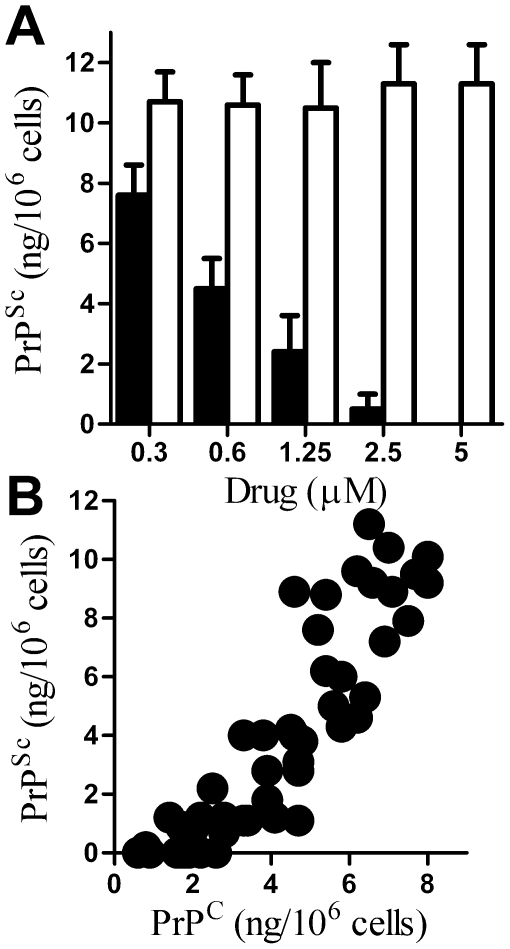
Glimepiride reduced the PrP^Sc^ content of ScGT1 cells. (A) The amount of PrP^Sc^ in ScGT1 cells treated for 7 days with different concentrations of glimepiride (▪) or with glibenclamide (□). Values shown are the mean average amount of PrP^Sc^ (ng/10^6^ cells) ± SD, n = 12. (B) Correlation between the amount of PrP^C^ at the surface of ScGT1 cells after 1 hour incubation with different concentrations of glimepiride and the amount of PrP^Sc^ in ScGT1 cells following treatment for 7 days.

**Figure 6:**
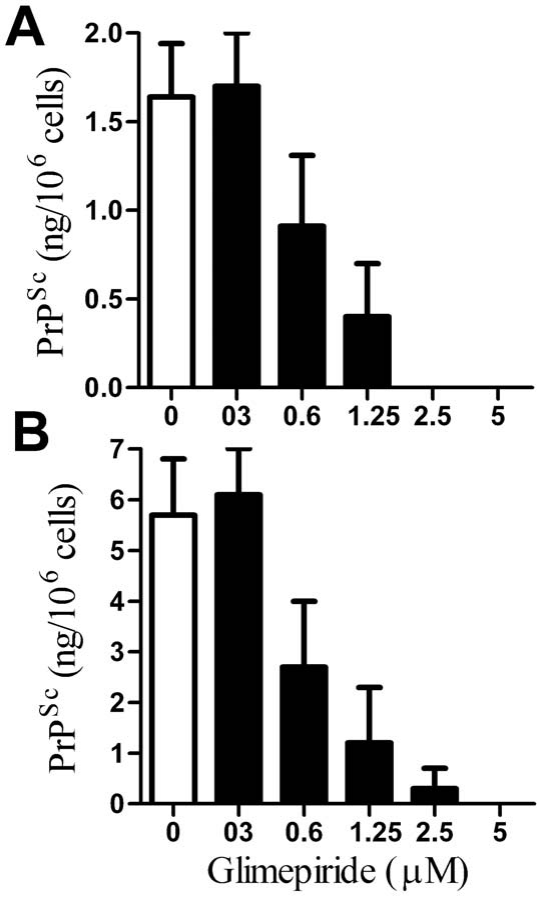
Glimepiride reduced the PrP^Sc^ content of prion-infected neuronal cells. (A) The amount of PrP^Sc^ in ScN2a cells following treatment for 7 days with control medium (□) or with different concentrations of glimepiride (▪). Values shown are the mean average amount of PrP^Sc^ (ng/10^6^ cells) ± SD, n = 12. (B) The amount of PrP^Sc^ in SMB cells treated for 7 days with control medium (□) or with different concentrations of glimepiride (▪). Values shown are the mean average amount of PrP^Sc^ (ng/10^6^ cells) ± SD, n = 12.

Since the cell surface is a possible site of the conversion of PrP^C^ to PrP^Sc^ [12], [13] the effect of glimepiride on the PrP^Sc^ content of ScGT1 cells was determined. Treatment with 5 µM glimepiride for 1 hour released PrP molecules from ScGT1 cells into the supernatant (8.4 ng/10^6^ cells). These PrP molecules were sensitive to digestion with 5 µg/ml proteinase K which reduced the PrP content to less than 0.05 ng/10^6^ cells, indicating that the PrP released was PrP^C^ or protease-sensitive PrP^Sc^. The PrP molecules were also sensitive to digestion with 50 µg/ml thermolysin (following digestion the PrP content of supernatants was reduced to less than 0.05 ng/10^6^ cells) indicating that the PrP released was PrP^C^ rather than protease-sensitive PrP^Sc^ [29]. Next, the effect of longer term treatment was examined. Twice daily treatment for 7 days with glimepiride, but not glibenclamide, caused a dose-dependent reduction in the amount of PrP^Sc^ in ScGT1 cells (Figure 5A). We were unable to detect PrP^Sc^ in ScGT1 cells treated with 5 µM glimepiride for 7 days. Moreover, these cells remained free of PrP^Sc^ when grown for a further month after the cessation of treatment. The amount of PrP^Sc^ in ScGT1 cells treated for 7 days with increasing concentrations of glimepiride showed a significant correlation with the amount of PrP^C^ released from the surface of glimepiride treated ScGT1 cells 1 hour after treatment, Pearson's coefficient = 0.868, P<0.01 (Figure 5B). This effect of glimepiride was not cell line specific as similar dose-dependent reductions in PrP^Sc^ content were observed in both ScN2a and SMB cells (Figure 6A and B).

Recent studies showed that glimepiride treatment of adipocytes released GPI anchored proteins in exosomes [19], [39]. Since PrP^Sc^ can also be released from prion-infected rabbit kidney cells in exosomes [34], the possibility that glimepiride might also stimulate exosome formation and the release of PrP^Sc^ from prion-infected neuronal cells was examined. The amount of PrP^Sc^ present in supernatants from ScGT1, ScN2a or SMB cells treated with 5 µM glimepiride for 7 days, was measured by ELISA. Treatment with glimepiride significantly reduced the amount of PrP^Sc^ in supernatants collected from ScGT1 cells (0.4 ng PrP^Sc^/10^6^ cells±0.3 compared with 1.97 ng±0.58, n = 9, P<0.01), SMB cells (0.3 ng PrP^Sc^/10^6^ cells±0.4 compared with 1.21 ng±0.28, n = 9, P<0.01) and ScN2a cells (0 ng PrP^Sc^/10^6^ cells compared with 0.45 ng±0.01, n = 9, P<0.01). These data indicated that the reduction of cell associated PrP^Sc^ observed after glimepiride treatment was not related to a stimulation of PrP^Sc^ release.

### Glimepiride treated neurones are resistant to PrP82-146 toxicity

**Figure 7:**
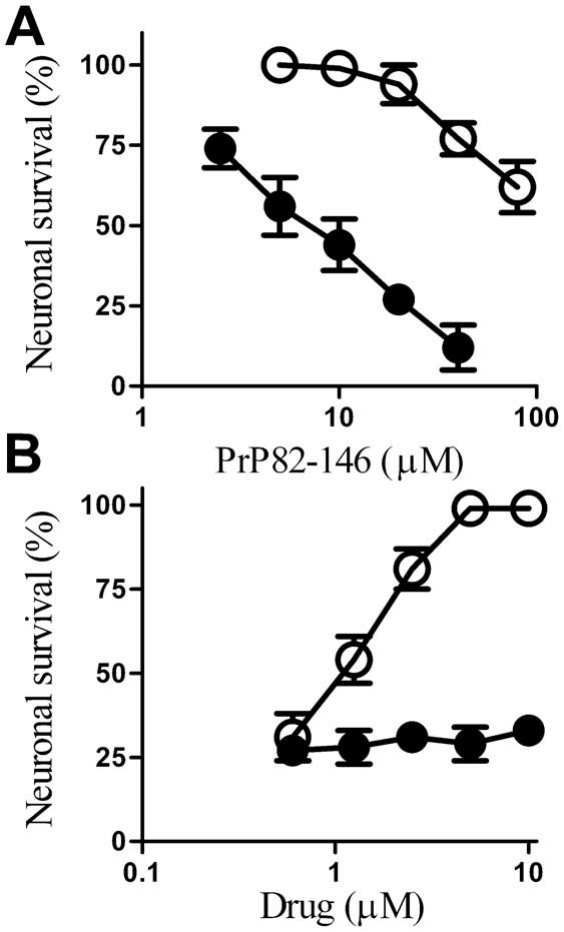
Glimepiride protects cortical neurones against PrP82–146. (A) The survival of cortical neurones pre-treated for 1 hour with control medium (•) or with 5 µM glimepiride (○) and incubated with varying concentrations of PrP82–146 for 5 days. Values shown are the mean average neuronal survival ± SD, n = 12. (B) The survival of cortical neurones pre-treated for 1 hour with varying concentrations of glimepiride (○) or glipizide (•) and incubated with 20 µM PrP82–146 for 5 days. Values shown are the mean average neuronal survival ± SD, n = 9.

**Figure 8:**
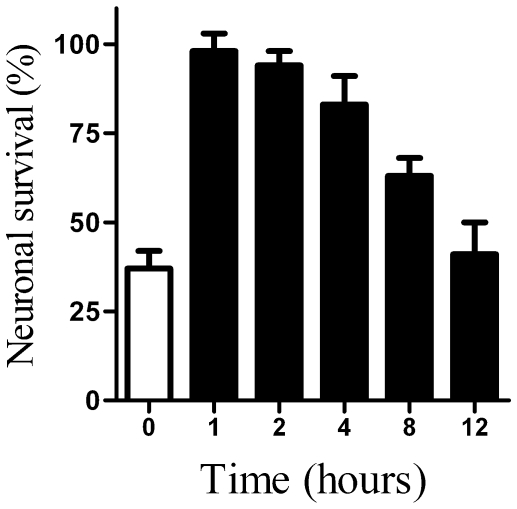
Protective effect of glimepiride is transient. The survival of vehicle treated cortical neurones (□) or cortical neurones pre-treated with 5 µM glimepiride for the time periods as shown (▪) and incubated with 20 µM PrP82–146 for a further 5 days. Values shown are the mean average neuronal survival ± SD, n = 12.

The addition of PrP82–146 reduced the survival of cortical neurones [23], [24]. Pre-treatment with 5 µM glimepiride protected cortical neurones against the toxic effect of PrP82–146 (Figure 7A). The concentration of PrP82–146 required to kill 50% of neurones (LD_50_) was at least 20 times greater, from 10 µM in mock-treated neurones to more than 200 µM PrP82–146 in glimepiride treated cells. The protective effect of glimepiride was dose-dependent (Figure 7B) and was not observed with glipizide. This effect of glimepiride was not specific for cortical neurones, the survival of hippocampal neurones incubated with 5 µM PrP82–146 was also significantly increased by pre-treatment with 5 µM glimepiride (24% cell survival±9 compared to 94%±8, n = 10, P<0.01). Glimepiride treated neurones were not resistant to all neurotoxins and did not protect against staurosporine (data not shown). The protective effect of glimepiride was dependent on pre-treatment and was not observed when 5 µM glimepiride was added 1 hour after addition of 20 µM PrP82–146 (41% cell survival±7 compared with 38%±5, n = 10, P = 0.4). It was also transient and was lost after 12 hours (Figure 8). The return of sensitivity to the toxic action of PrP82–146 coincided with the return of PrP^C^ to the surface of the neurones as shown in Figure 2.

**Figure 9:**
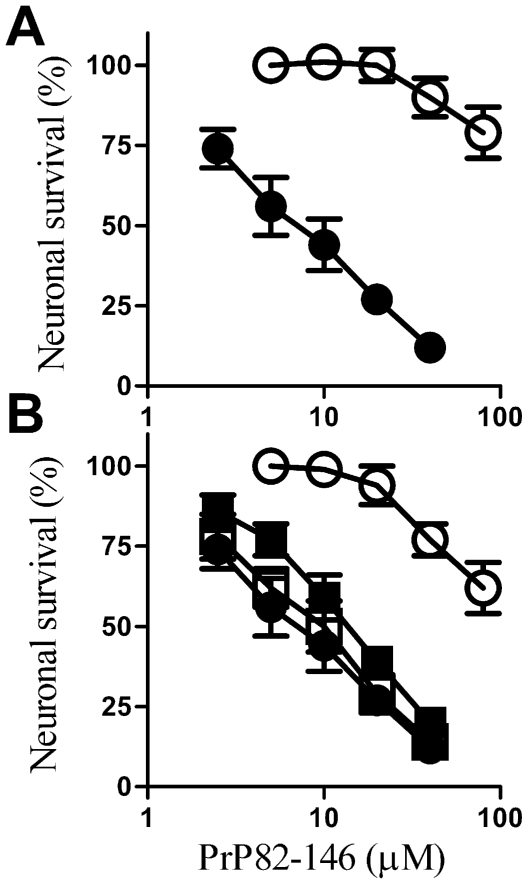
Digestion with PI-PLC protects neurones against PrP82–146. (A) The survival of cortical neurones pre-treated for 1 hour with 0.2 units PI-PLC/ml (○) or with control medium (•) and incubated with varying concentrations of PrP82–146 as shown. Values shown are the mean average neuronal survival ± SD, n = 9. (B) The survival of cortical neurones pre-treated for 1 hour with a vehicle control (•), with 5 µM glimepiride (○), with 500 µM p-CMPS (□) or with a combination of 5 µM glimepiride and 500 µM p-CMPS (▪) and incubated with varying concentrations of PrP82–146. Values shown are the mean average neuronal survival ± SD, n = 9.

Since glimepiride activates an endogenous GPI-PLC [16], [17] the effects of PI-PLC on neuronal responses to PrP82–146 were also examined. Pre-treatment with 0.2 units PI-PLC/ml was found to increase the survival of cortical neurones subsequently incubated with PrP82–146 (Figure 9A). Next we tested the hypothesis that the protective effect of glimepiride was due to activation of an endogenous GPI-PLC. First we showed that pre-treatment with a GPI-PLC inhibitor (500 µM p-CMPS) did not affect the survival of neurones subsequently incubated with PrP82–146. Next, we showed that the protective effect of 5 µM glimepiride was reversed by the inclusion of 500 µM p-CMPS indicating that protection was dependent upon activation of an endogenous GPI-PLC (Figure 9B).

### Glimepiride reduced the binding of PrP82–146 by neurones

**Figure 10:**
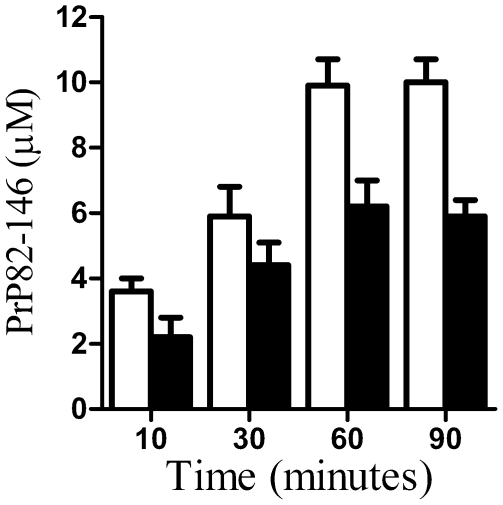
Glimepiride reduced the binding of PrP82–146 to neurones. The amount of PrP82–146 in cell extracts from cortical neurones pre-treated for 1 hour with a vehicle control (□) or with 5 µM glimepiride (▪) and exposed to 10 µM PrP82–146 for different times periods as shown. Values shown are the mean average amount of PrP82–146 (µM) ± SD, n = 9.

Since PrP^C^ acts as a receptor for PrP peptides [40], the effect of glimepiride on the binding of 10 µM PrP82–146 to cortical neurones was examined. The amount of PrP82–146 bound was time-dependent over 60 minutes. Neurones pre-treated with 5 µM glimepiride bound significantly less PrP82–146 than mock-treated neurones (Figure 10). It was noted that glimepiride treatment did not completely block the binding of PrP82–146 to neurones. Thus, 60 minutes after the addition of 10 µM PrP82–146, glimepiride treated neurones bound 6.2 nM PrP82–146±0.8 compared to 9.9 nM PrP82–146±0.8 in controls (n = 8). Similar results were obtained when neurones were pre-treated with 0.2 units PI-PLC/ml, which after 60 minutes had bound 5.3 nM PrP82–146±0.7, n = 8.

### Glimepiride reduced activation of cPLA_2_ by PrP82–146

**Figure 11:**
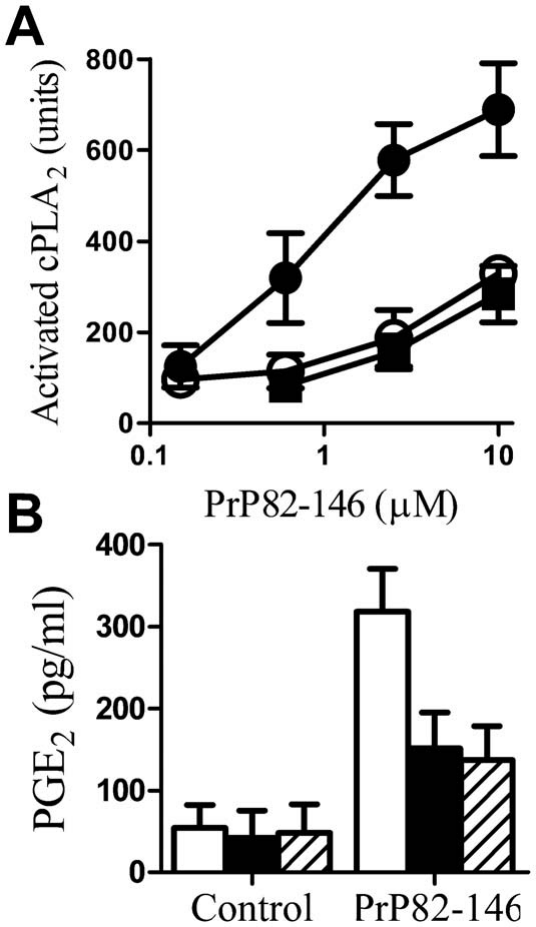
Glimepiride reduced the activation of cPLA_2_ by PrP82–146. The amount of activated cPLA_2_ in cell extracts from cortical neurones pre-treated for 1 hour with a vehicle control (•) 5 µM glimepiride (○) or PI-PLC (▪) and incubated with varying concentrations of PrP82–146 for 24 hours. Values shown are the mean average amount of activated cPLA_2_ (units) ± SD, n = 12. (B) The amount of PGE_2_ in cell extracts from cortical neurones pre-treated for 1 hour with control medium (□), 5 µM glimepiride (▪) or PI-PLC (striped bars) and incubated with control medium or 10 µM PrP82–146. Values shown are the mean average amount of PGE_2_ (pg/ml) ± SD, n = 9.

Unregulated activation of PLA_2_ is recognized as a key event in some neurodegenerative diseases [41], [42], [43]. The activation of PLA_2_ is the first step in the production of eicosanoids, docosanoids and platelet activating factors, high concentrations of which cause glial cell activation, synapse degeneration and neuronal death. The addition of PrP82–146 increased the amount of activated cPLA_2_ in neurones. Treatment with 5 µM glimepiride alone did not alter the amount of activated cPLA_2_ in cortical neurones (100 units activated cPLA_2_±8 compared to 97±10, n = 9, P = 0.4), but greatly reduced the activation of cPLA_2_ induced by PrP82–146 (Figure 11A); treatment with 5 µM glibenclamide or 5 µM glipizide had no effect. Digestion of neurones with PI-PLC also reduced activation of cPLA_2_ induced by PrP82–146. To confirm the effect of glimepiride on PrP82–146 induced PLA_2_ activation, neuronal PGE_2_ production was also measured. As would be predicted, PGE_2_ production from neurones pre-treated with 5 µM glimepiride, or with PI-PLC, was significantly lower than that of untreated neurones after incubation with 10 µM PrP82–146 for 24 hours (Figure 11B).

## Discussion

In this study we report that treatment with glimepiride reduced the amount of PrP^C^ expressed at the surface of primary cortical neurones, neuronal cells and prion-infected neuronal cells. Subsequently, glimepiride treatment significantly reduced the amount of PrP^Sc^ within 3 prion-infected neuronal cell lines (ScGT1, SMB and ScN2a cells) and glimepiride treated cortical neurones showed increased resistance to the toxic effects of PrP82–146. The protective effect of glimepiride was accompanied by reduced binding of PrP82–146 to neurones, reduced activation of cPLA_2_ and PGE_2_ production. These effects of glimepiride were observed at physiological concentrations [44], however they were not shared by 2 other sulphonylureas used to treat diabetes, glibenclamide or glipizide.

The cellular location of PrP^C^ is controversial and although PrP^C^ is expressed at the cell surface, it is also found within cells [45], [46]. Our findings support the view that a proportion of the PrP^C^ in cortical neurones is in an intracellular pool, since it resisted both digestion with PI-PLC and biotinylation with a cell impermeable biotin-conjugate. Treatment of cortical neurones, neuronal cells and prion-infected neuronal cells with glimepiride reduced the amount of PrP^C^ expressed at the cell surface and caused its release into the supernatant whereas the amount present in cell extracts was reduced by about a third. The release of PrP^C^ into supernatants may occur as a consequence of exosome formation, or following digestion by phospholipases and proteases [11], [34], [35], [36], [37]. Glimepiride activates an endogenous GPI-PLC in adipocytes [18] and many of its effects on neurones were replicated by the addition of PI-PLC. In addition, glimepiride induced release of PrP^C^ was reversed by the inclusion of p-CMPS, an inhibitor of GPI-PLC. Whereas PrP^C^ bound to C18 columns, PrP^C^ released from glimepiride treated cells did not bind, consistent with the loss of hydrophobic acyl chains. Why glimepiride affects PrP^C^ at the surface of neurones, but not intracellular PrP^C^, is unclear. It is possible that glimepiride does not penetrate the cell, an alternative explanation may be that GPI-PLC is associated with cell surface PrP^C^, but is absent from intracellular stores of PrP^C^.

Glimepiride treatment did not affect all GPI-anchored proteins. Thus, while digestion of neurones with PI-PLC released several GPI-anchored proteins into the supernatant, including PrP^C^ and Thy-1, glimepiride treatment released PrP^C^ but not Thy-1 (unpublished data). This specific effect of glimepiride may be due to the activation of an endogenous GPI-PLC that is closely associated with specific GPI-anchored proteins such as PrP^C^, but not with others including Thy-1 or CD55 which occupy separate domains upon the cell surface [47]. An alternative explanation is that CD55 and Thy-1 may contain GPI anchors that are resistant to endogenous GPI-PLC.

The precise cellular location in which PrP^C^ is converted to PrP^Sc^ remains controversial with advocates for the endosomal recycling compartment [48], [49]. However, anti-PrP antibodies reduced PrP^Sc^ formation, suggesting that conversion occurs either at the cell surface, or after PrP^C^ has been internalised from the cell surface [13], [15], [50]. Such observations indicate that surface expression of PrP^C^ is a prerequisite for PrP^Sc^ formation and that glimepiride reduced PrP^Sc^ formation by shedding PrP^C^ from the cell surface. Our finding that glimepiride treatment released PrP molecules from ScGT1 cells raised concerns that glimepiride might cause the release of PrP^Sc^ and facilitate its spread throughout the brain. However, all PrP released from cells within 1 hour of treatment was sensitive to digestion with proteinase K. Although the presence of proteinase K sensitive PrP^Sc^ is well documented [51], the released PrP was also sensitive to digestion with thermolysin which has been reported to digest PrP^C^ but not PrP^Sc^ [29]. Collectively these results indicate that the PrP released from ScGT1 cells was PrP^C^. The longer term effects of glimepiride treatment showed that twice daily treatment for 7 days caused a dose-dependent reduction in the PrP^Sc^ content of ScGT1, ScN2a and SMB cells. It also reduced the amount of PrP^Sc^ released into supernatants over this period, excluding the possibility that the reduction in cell-associated PrP^Sc^ was due to glimepiride induced the release of PrP^Sc^ from cells. Our findings are consistent with the hypothesis that glimepiride acts by limiting the supply of PrP^C^ to cellular sites that are essential for PrP^Sc^ formation.

Glimepiride treatment of cortical neurones affected cell surface PrP^C^ but not intracellular PrP^C^. Similar results were obtained with GT1 and ScGT1 cells in which approximately 70% of PrP^C^ molecules remained after treatment with glimepiride or PI-PLC and about the same amount resisted labelling by membrane impermeable biotin. These results suggest that PrP^Sc^ is formed from a subset of PrP^C^ molecules that recycle to and from the cell surface. Perhaps more significantly they indicate that the intracellular PrP^C^ molecules were poor substrates for conversion to PrP^Sc^. In addition, the repopulation of surface PrP^C^ in glimepiride treated cells was from newly synthesised PrP^C^ rather than from the intracellular pool, as it was delayed by the inclusion of the protein synthesis inhibitor cycloheximide. We conclude that there are at least 2 pools of PrP^C^: one that consists of PrP^C^ molecules that recycle to and from the cell surface and are susceptible to conversion to PrP^Sc^ and another pool of PrP^C^ molecules that are mostly intracellular and are not readily converted to PrP^Sc^ suggesting that they follow different trafficking pathways. Such results are consistent with reports that altering the trafficking of PrP^C^ alters PrP^Sc^ formation [52], [53].

Pre-treatment with glimepiride protected cortical and hippocampal neurones against the toxic action of PrP82–146; it increased the LD_50_ of PrP82–146 by more than 20 fold, from 10 µM to over 200 µM. However, glimepiride treated neurones remained sensitive to staurosporine (data not shown). The protective effect of glimepiride required pre-treatment; glimepiride did not rescue neurones that had been incubated with PrP82–146 for 30 minutes indicating that it affected an early event in PrP82–146 induced toxicity. Moreover, the protective effect of glimepiride was transient. Restoration of PrP^C^ to the surface of neurones following glimepiride treatment was associated with their increased sensitivity of neurones to PrP82–146. Neurones treated with PI-PLC were also protected against PrP82–146 suggesting that glimepiride-mediated neuroprotection was due to activation of GPI-PLC. This hypothesis was strengthened by the finding that the protection induced by glimepiride was reversed following the addition of the GPI-PLC inhibitor p-CMPS.

PrP^C^ has been proposed as a receptor for PrP peptides [40] and glimepiride reduced the binding of PrP82–146 to neurones. It is worth noting that although treatment with glimepiride or PI-PLC significantly reduced the binding of PrP82–146 to neurones, these cells still bound significant amounts of PrP82–146 (about 50% of the binding of untreated cells) indicating that PrP82–146 can bind to neurones via a PrP^C^ independent mechanism. Many proteins have been proposed to be prion receptors [54] and it seems likely that glimepiride treated cells express some of them, or that PrP82–146 binds to cells independently of specific protein interactions. Since the reduction in PrP82–146 binding alone did not fully explain the protective effects of glimepiride, the effects of glimepiride on PrP82–146 induced cell signalling were examined. The unregulated activation of PLA_2_ is recognized as a key event in some neurodegenerative diseases [41], [42] and is the first step in the production of eicosanoids, docosanoids and platelet activating factors, high concentrations of which can cause glial activation, synapse damage and neuronal death. Furthermore, PLA_2_ plays a critical role in neurotoxicity caused by PrP peptides [55]. Our experiments showed that PrP82–146 activated cPLA_2_ in neurones and increased PGE_2_ production, a marker of PLA_2_ activation that is increased in scrapie infected mice [56], [57] and in the cerebrospinal fluid of patients with CJD [58], [59]. Pre-treatment with glimepiride significantly reduced PrP82–146 induced activation of cPLA_2_ and the production of PGE_2_. Although the precise mechanism is not clear, the activation of cPLA_2_ occurs in cholesterol-sensitive lipid rafts [60], [61]. Since glimepiride induced digestion of GPI anchored proteins affects membrane cholesterol in adipocytes [62] our results are consistent with the hypothesis that glimepiride modifies lipid rafts required for cPLA_2_ activation. Other studies suggest that the neurotoxicity of PrP peptides is through the amplication of PrP^C^ associated signalling pathways [63] which may be downregulated following glimepiride treatment.

Prion infection increased cholesterol in cell membranes [32]. Since the insulin receptor is found within lipid rafts [64] and insulin signalling is cholesterol dependent [65], [66], prion infection induced changes in cell cholesterol may modify insulin signalling. This is consistent with observations that prion infection affects insulin and insulin-like growth factor receptors in cell lines [67], [68] and that scrapie infection induced diabetes mellitus in hamsters is directly damaging the central nervous system, without affecting the pancreas [69]. Thus, glimepiride treatment may also reverse prion-induced effects on insulin signalling.

One important consequence of the effect of glimepiride on PrP^C^ may be of relevance to the treatment of Alzheimer's disease. PrP^C^ was identified as a receptor that mediated the impairment of synaptic plasticity induced by amyloid-β oligomers [70]. Thus, the pharmacological regulation of PrP^C^ by glimepiride could provide a new approach to the inhibition of amyloid-β mediated synapse damage in Alzheimer's disease patients.

New approaches to the treatment of neurodegenerative conditions including prion diseases are urgently required. We have demonstrated that treatment of neurones with glimepiride caused the shedding of PrP^C^ from the cell surface through activation of an endogenous GPI-PLC. Treatment reduced the formation of PrP^Sc^ in prion-infected neuronal cell lines and increased the survival of neurones incubated with PrP82–146. Glimepiride treatment reduced both the amount of PrP82–146 ingested by neurones and PrP82–146 induced activation of cPLA_2_. Our results suggest that glimepiride could prove beneficial in the treatment of prion diseases.
